# Pilot of a Charter to Improve Management of Medicines and Oral Care for Residents with Dysphagia in Care Homes

**DOI:** 10.3390/geriatrics3040078

**Published:** 2018-11-15

**Authors:** Jacqueline E. Morris, Frances Hollwey, Dharinee Hansjee, Rachel A. Power, Richard Griffith, Timothy Longmore, David G. Smithard, Eleanor Dann-Reed, David J. Wright

**Affiliations:** 1 Patients Association, Harrow HA1 3YJ, UK; jacqueline.seifert.morris@gmail.com (J.E.M.); frances.hollwey@patients-association.com (F.H.); rachel.power@patients-association.com (R.A.P.); 2 Royal College of Speech and Language Therapists, London SE1 1NX, UK; dharinee.hansjee@nhs.net; 3 College of Human and Health Sciences, Swansea University, Singleton Park, Swansea SA2 8PP, UK; richard.griffith@swansea.ac.uk; 4 Elvington Medical Practice, Elvington, York YO41 4LD, UK; timothy.longmore@nhs.net; 5 Lewisham and Greenwich NHS Trust, Queen Elizabeth Hospital, London SE18 4QH, UK; david.smithard@nhs.net; 6 School of Pharmacy, University of East Anglia, Norwich Research Park, Norwich NR4 7TJ, UK; E.Reed@uea.ac.uk

**Keywords:** dysphagia, care home, covert administration, charter

## Abstract

Research in care homes has demonstrated that medication management practices in patients with dysphagia and those receiving medicines covertly may be inappropriate, illegal, and potentially cause harm. This paper presents the results of a feasibility study piloting a resident and healthcare professional best practice charter to improve such practices in care home residents with dysphagia. A charter was developed through a multi-professional expert panel, implemented in one care home, and then piloted in 22 homes in England, Wales, and Northern Ireland. A website was setup and developed iteratively to support the process. Care home staff and residents provided initial feedback on the implementation process and on perceived outcomes six months post implementation. A total of 16 (88.9%) out of 18 respondents from nine homes for six months reported a positive response to the charter. More than 80% of responses regarding the implementation process, impact on staff confidence, and perceived usefulness of the charter were positive. Perceived effectiveness and usefulness could, however, be further improved especially the perceived effect on frequency of medication review, which is reliant on external stakeholder involvement. The charter and supporting website were well received with respondents believing that it was useful, staff showing more confidence, and residents having enhanced care. Approaches to enhancing the charter’s effectiveness were identified.

## 1. Introduction

In 2011, there were 291,000 older people aged 65 and over living in care homes in the UK and, of these 164,000 people, were ones over 85 years of age [1]. They have complex needs including significant frailty, dementia, and disability [2]. Researchers in the UK have shown that care home residents are prescribed a mean of 7.2 medications and that seven out of 10 residents were found to have been exposed to at least one medication error [3]. These were not limited to drug, dose, and formulation selection but also included dispensing, administration, and monitoring errors. Consequently, interventions to reduce the likelihood of medication-related errors in care homes were identified as being required [3]. Researchers using standardized criteria continue to identify high proportions of prescribed medicines in care homes as being potentially inappropriate [4]. While inappropriate antipsychotic prescribing is a common theme [5,6], benzodiazepines, laxatives, antidepressants, and antibiotics have all been shown to be prescribed more frequently in this environment [7]. Regular medication review is the most commonly reported intervention used to improve prescribing in care homes and evidence demonstrates that it can effectively reduce the number of inappropriate medicines prescribed [8].

It has been proposed that most prescribing decisions are influenced by the care home staff since they initiate the majority of requests for medicines [9] and, therefore, interventions, which are focused on care home staff that may also improve medicine use. With the high turnover of staff within care homes [10,11] and poor culture of innovation and change activities (apathy/lack of engagement and champions) [11] and external forces (primary care and prescribing advisors), it is difficult to both instill and retain knowledge of good practice within care homes and, therefore, to effect meaningful ongoing practice change. To encourage change in organizational culture, there needs to be a critical mass of change champions and, in the case of care homes, this will include all people involved in the prescribing/dispensing pathway. It is critical that values and goals are shared widely to encourage empowerment for staff, residents, and families [11]. Charters have been reported to be introduced in care homes to improve the care of residents and, while they are perceived to be important and necessary with limited awareness, their impact is likely to be minimal [12].

Dysphagia is also common within this environment with an estimated prevalence of 7% to 40% depending on the criteria used for the assessment and resident population focus [13]. There are multifactorial reasons for the high prevalence including the loss of muscle strength and elasticity within the mouth, pharynx, and supra-hyoid muscles, which result from frailty and sarcopenia and, secondarily, to stroke, neurodegenerative diseases, and dementia [14]. Given the multifactorial etiology and the association of dysphagia with functional decline and increased mortality, it is now considered a geriatric syndrome. In the presence of dysphagia, there is an increased risk of aspiration pneumonia [15,16,17], reduced nutritional [18], and hydration status [19] and quality of life [20]. It is, therefore, important that it be identified at an early stage to enable food, drink, and medication administration to be modified accordingly.

Oral pathogens are the likeliest cause of pneumonia. Therefore, good oral care is essential to reduce the risk of pneumonia [21,22]. Failure to deliver good mouth and oral care can contribute to difficulties with swallowing and exacerbate dehydration, malnutrition, and frailty [23]. Education of nurses and caregivers has been identified as a necessity [24] with deficits in nurse knowledge regarding the link between oral hygiene, dysphagia, and pneumonia identified [25]. 

Ill health, frailty, and swallowing in older people will be exacerbated by the side effects of medicines [26] particularly those with anticholinergic effects since they are known to cause dry mouth [27]. Recent research has found that care home residents with dysphagia were more than twice as likely to experience medication errors compared to those without [28]. This was largely due to the added complexity of identifying the most appropriate formulation or method of administration when tablets or capsules may no longer be appropriate rather than problems with the drug or dose selection. Due to the number of unreported signs of aspiration observed during the research process, the authors recommended that nurses and care staff should be trained to identify signs and symptoms of dysphagia to ensure that the condition is identified in a timely manner, which reduces associated medical complications [26,28]. 

In the presence of dysphagia, caregivers frequently resort to tablet crushing, capsule opening, and mixing with food to ease administration [29,30]. The disguising of medication in food or drink has been reported to occur in 43% to 71% of nursing homes [31] and often takes places without documentation and consultation with all relevant parties. Whenever medicines are mixed in food, it is important that either the resident is made aware and is in agreement of it or that appropriate procedures are implemented to ensure that appropriate safeguards are in place. Training of care home staff and the implementation of audits have been recommended to address this [31].

In response to reported concerns regarding the administration of medicines to residents in care homes with respect to tablet crushing, covert administration, medication errors, and inappropriate use of medicines, the Patients Association’s in the UK undertook a survey of “medicines related care of residents with dysphagia in care homes”. The relatively small-scale study found that only 10% of the homes had a specific protocol to guide staff in administering medicines to people with dysphagia and only 20% had arranged training in this important area [32].

Using the results of the survey as a driver for change in care home practices, an expert panel was convened and chaired by the Patients Association to address these concerns. Evidence demonstrated the need for more frequent medication review, the need to identify residents with dysphagia more effectively, and greater care home staff training especially regarding the laws surrounding covert administration and the dangers of tablet crushing and capsule opening. To protect residents from pneumonia, care home staff also require training in the provision of good oral care and, to optimize decisions, it is necessary to involve the resident or, if they don’t have the capacity themselves, it is necessary to receive support from all appropriate members of the multi-disciplinary team.

The panel consequently developed best practice charters (referred to as charter(s) for brevity in the rest of the paper) based on expert consensus derived from the National Institute of Health and Clinical evidence (NICE) for residents and caregivers in care homes and a strategy for implementation. The charters were designed to encompass the principles by which care homes would agree to practice and provide care to their residents. While not legally enforceable, they could be used to demonstrate the home’s commitment to the provision of high quality care. The aim of this paper is, therefore, to describe the results from piloting the charter in a small number of homes in England, Wales, and Northern Ireland.

## 2. Materials and Methods 

The project was deemed to be a quality improvement service evaluation by the University of East Anglia Faculty of Health ethics committee and, therefore, formal ethical approval was not sought.

### 2.1. Charter and Related Website Development

**Figure 1:**
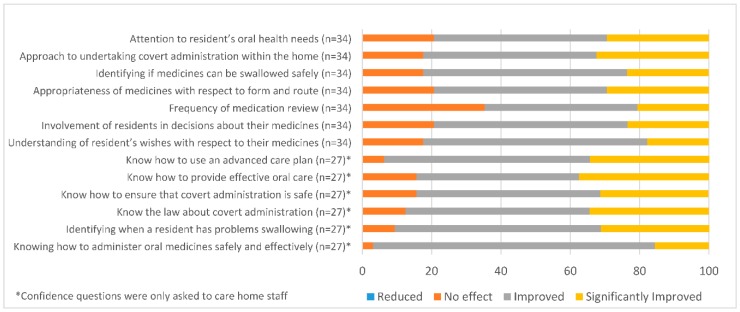
Reported effect of confidence of charter on care home staff and perceived impact of charter on both care home staff and residents.

An expert panel was convened consisting of senior care home managers and nursing representatives, primary and secondary care medical practitioners with interest and expertise in care of the older person, national speech and language therapy expert, pharmacists with expertise in dysphagia management, an expert in healthcare law, and representatives from the Patients Association. The charter, which was informed by the national guidance on improving medicines management in care homes [33], was drafted by the expert panel with statements regarding the need for regular medication review to identify swallowing deficits, ensure that medicines are administered appropriately, ensure that the laws related to covert administration were adhered to, and all decisions should involve the resident or their representative. Versions were created both for residents and caregivers and revised iteratively over three meetings whereby stakeholders had been engaged in-between at feasibility and piloting phases until a final version was agreed (Figure 1).

To effectively communicate the charter, a website www.carehomecharter.org was set up containing both charters and short videos by different experts explaining each of the statements with respect to rationale and how to adopt them [34]. Each charter is also introduced by two experts in the field so that users can find out why their peers believe that it is important and how it was developed. Additionally, links to supporting materials, websites, and expert bodies were provided.

### 2.2. Feasibility Testing

**Figure 2:**
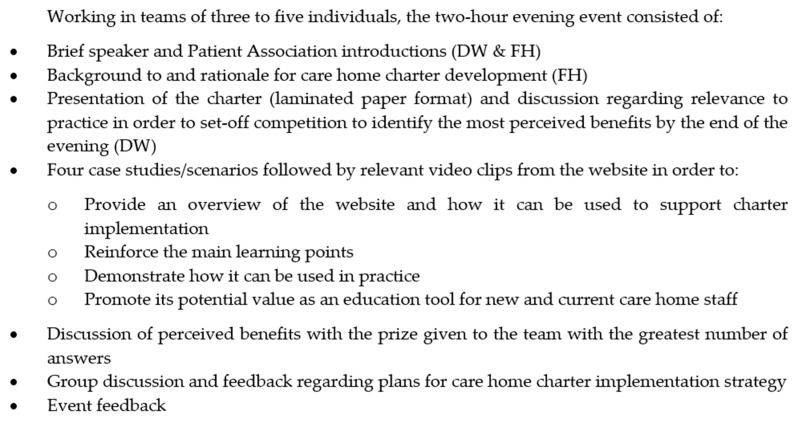
Care home charter launch and training event plan.

The first version of the care home charter and website were presented to care home staff within a bespoke two-hour meeting to obtain feedback on both charter content and how best to encourage its implementation in practice. Recommendations from this were then used to revise the website and create the plan for piloting implementation of the charter. It was agreed that homes in the pilot locations would be invited to participate in a 2-h care home charter launch and training event and that this should be held away from the workplace and in the presence of staff from other homes to enable the sharing of ideas (Figure 2). It was also agreed that both a senior and junior member of staff and an optional additional third person deemed to have an interest in medicine management within each of the homes would be invited to become charter champions.

Guidance and examples of how to incorporate the charter into electronic care plans and routine audits were provided and uploaded onto the website for sharing with other homes. It was also agreed that a checklist on how to identify possible dysphagia, created by our speech and language expert, would be helpful. This was also added [35]. At this stage, links to useful websites and guidelines were also included in the website.

### 2.3. Feasibilty (Pilot) Methods

In addition, 10 care homes in each of Northern Ireland and North Wales and 20 care homes in London, identified through gatekeepers within the expert panel, were approached to take part in a pilot of the charter. The expert panel purposively chose to locate the pilot in three different countries to enable any differences in local policies to be identified.

At the end of each of the launch and training events, undertaken January 2018, all attendees were asked to complete feedback on the training and event and were informed that they would be sent a questionnaire at three months for feedback on the implementation process. A further questionnaire was sent at six months to capture feedback from staff and residents regarding the perceived impact of the charter. The primary aim of these questionnaires was to enhance the implementation process iteratively and to test the impact questionnaires for both the response rate and face validity. Content validity for all questionnaires was derived from expert panel feedback at each stage of the process.

The questionnaires, which were designed and revised by the expert panel, consisted of less than 40 questions in which the majority were closed and request a response on a five point Likert scale. A small number of open questions were included to obtain explanations for responses and to enable respondents to provide additional information, which they believed may be useful or relevant.

The training event questionnaire, which was given to all attendees, sought feedback on overall assessment and whether it met personal objectives or expectations and whether it would be useful for implementation and application purposes. Additionally, advice was sought on what could be done differently or additionally to enhance the likelihood of the implementation success.

The implementation questionnaire, which was sent for completion by care home staff only, sought information on how the charter had been implemented, the initial response, barriers, and enablers to its effectiveness and feedback on the website as a support tool. Four questionnaires were posted to each home with a request that two were given to senior staff and two to junior staff for completion. Questions regarding the perceived impact were additionally included to inform the design of the six-month questionnaire.

The impact questionnaire was based around perceived effectiveness regarding meeting the care home charter objectives (Figure 1). Multiple versions were created for senior and junior care staff plus residents and their relatives/families. The questions were similar but were modified for different recipients. Two copies of each questionnaire were sent to each pilot home with a stamped addressed envelope with the home name on it to enable follow up. Individual questionnaires were anonymous. The Patients Association held the names of the care homes and managers and was responsible for posting out all surveys, which were then returned to the University of East Anglia for analysis. The care home managers were used as gatekeepers at both stages and selected who to give the questionnaire to for completion.

Questionnaires were designed and implemented to maximize response rates [36]. A postal survey was used since this was identified as the preferred method during the initial design phase. The questionnaire was kept to a minimum length, a financial incentive was provided for completion, the university and Patients Association logos were placed on the questionnaire, and confidentiality was assured. Staff were pre-notified of the questionnaire at the training event and a reminder e-mail was sent prior to posting and again within two weeks of initial posting. For the impact questionnaire, non-respondent homes were also telephoned after one month.

Data analysis was largely descriptive with content analysis performed on all responses to open questions. Results were presented to the expert panel at each stage in order to agree on the next steps.

## 3. Results

### 3.1. Launch and Training Event

A total of 11 (55%) care homes from London, 8 (80%) care homes from North Wales, and 3 (30%) care homes from Northern Ireland attended the pilot launch event. Participants included 27 care home managers/deputy managers, 6 senior caregivers including nurses, 4 healthcare assistants, and 4 pharmacists and pharmacy support staff.

Regarding overall assessment of the event, 28 (76%) care homes rated the event as excellent and 8 (22%) care homes rated the event as good with one declining to comment. In addition, 36 (97%) agreed that the event achieved its objectives, met their knowledge and information expectations, and would be useful for implementation. The same individual 1 (3%) disagreed with all statements and reported that GP involvement would have enhanced it.

The elements that they reported to be most useful were having tools available to use, information on the website, pharmacy input, case scenarios and discussion, use of video clips to give the charter information, involvement of a multidisciplinary team in the implementation process, on line training for staff to access, and sharing knowledge with colleagues.

The event could have been improved with more time, more group activity, and more time spent explaining the audit tool and using more case studies.

### 3.2. Pilot Implementation Feedback (3 Months Post-Implementation)

A total of 18 individuals returned the implementation questionnaire from at least 9 (40.9%) of the 22 different homes. In addition, five individuals did not report the care home from where the questionnaire was sent. The main methods of implementation were by introducing an agenda point at a routine meeting (11 (61.1%)), prominent display (10 (55.6%)), and by handing out paper copies (9 (50%)). Two (11.1%) homes reported organizing a bespoke care home meeting and 3 (16.7%) created a specific staff bulletin. Other methods reported were via individual meetings, by creating a specific folder, and through discussions at handovers.

In addition, 16 (88.9%) reported a positive reception with 2 (11.1%) stating it received a mixed reception. However, in both cases, this was because staff believed that they were already adhering to all standards within the charter and, therefore, it would have minimal effect.

The main message regarding how to make it more effective were to ensure that the charter was included in staff inductions and as part of ongoing training. Conversely, the main barriers to its effectiveness were seen as a lack of staff awareness and routine monitoring and review. A barrier that was identified by a small number of respondents was a lack of buy-in particularly by the GP. Implementation could be improved by training GPs, the multi-disciplinary team, and care home staff. Respondents were very positive regarding all elements of the charter and website with no negative responses.

Following review of the feedback, the expert panel agreed that the launch event training materials and answers should be made available on the website for care home staff to use to train their staff either in bite size pieces or as a whole package. It was also agreed that an on-line quiz should be developed that tested whether someone had read the charter and viewed the website, which would then provide an automatic certificate stating that the individual had committed to the charter. This was to be provided free of charge to ensure that the finances did not create a barrier to access. Lastly, due to the multitude of tools that were now available on the website to enhance implementation, a guide to successful implementation for care home managers was provided. The availability of all tools was relayed to all pilot homes two months prior to the final questionnaire.

### 3.3. Pilot Impact Feedback (Six Months Post-Implementation)

Questionnaires were returned from 9 (40.9%) homes including 18 (37.5%) from senior care staff, 18 (37.5%) from junior care staff, and 12 (25.0%) from residents or relatives.

Table 1 provides a summary of how the charter had been implemented and the website used by the different respondents. Six (54.5%) residents and relatives were aware that the charter had been implemented within their home. The number of responses available for each question differed and, where this is occurred, n is stated on the left hand side.

Positive comments reported by respondents included:

‘*This training has been useful. It has made me more aware of how important it is that I involve the multidisciplinary team before making the covert medication decision*.’ (Senior Cargiver)

‘*Very useful information for daily working*.’ (Senior Cargiver)

‘*Being able to identify residents with swallowing difficulties and making referrals to the SALT* [Speech and Language Therapist] *has ensured safe administration of medication to people with swallowing difficulties*.’ (Junior Caregiver)

One junior caregiver conversely explained how they were ‘*not sure about the charter, as has not been introduced to that but, I am confident in all of the above* [confidence statements]’.

Thirty junior and senior caregivers (83.3%) provided the date of the last time they had been formerly trained in medicine administration. One (3.3%) had been trained in 2016, 8 (26.7%) were trained in 2017, and 21 (70.0%) were trained in 2018. Fourteen (77.8%) of senior caregivers and 15 (83.3%) of junior caregivers stated that they regularly undertook medicine administration duties.

Table 2 provides a summary of the respondents’ thoughts regarding the overall usefulness of the charter for both their role and on the quality of care for residents.

## 4. Discussion

This paper describes the process undertaken to pilot and test the implementation of a charter to improve care for residents with swallowing problems in care homes. The charter was placed on an open access website with relevant links to relevant training tools. Collaboration with a care home team to develop the implementation strategy was effective and the tools provided and recommended were reported to be well utilized. Similar utilization was noted with the tools developed and implemented during the pilot phase. Those tools that required registration and payment for access e.g., NEWT guidelines for administration of medication to patients with enteral feeding tubes or swallowing difficulties, or significant time to complete e.g., On-line course in dysphagia, were less frequently reported as being used, which aligns with expectations.

Implementation of the charter improved staff confidence in all of the stated areas including knowledge of how to identify swallowing problems, use of advanced care plans, administration of medicine, covert administration, and the laws underpinning this. Although most respondents believed that it improved their knowledge regarding the law surrounding covert administration and advance care decisions, they were negative regarding these elements and this may be explained by the complexity of the subjects.

Most respondents believed that the charter would increase involvement of residents in decision making, improving oral health, enhancing identification of swallowing problems, and improving the administration of medicine. The lowest response whereby just over half of respondents were positive was the frequency of medication review. The review of prescribed medications are accepted as the role of the pharmacist or general practitioner with care home staff taking a passive role or reporting that concerns are ignored. The medication review needs to involve in residents/families/staff and GPs or pharmacists. Perhaps the time has come to encourage care homes to be more proactive and primary care staff to listen to them. 

While regular training in medication administration was reported to be routinely undertaken by the respondents, the charter, which is specific to medication review and administration of medicine to residents with dysphagia, has improved confidence in the area. Training in medication administration is not a mandatory requirement for care home staff who do not undertake this role and the pilot gave no insight into the perceptions of other care staff regarding its implementation. For the charter to be fully effective, staff in all care homes need to be aware of it especially those areas for identification of dysphagia, promotion of good oral care, and involving residents in all decisions regarding them.

Responses from staff were largely positive, describing the charter as ‘useful’ for improving resident medicine management and oral care rather than ‘very useful’. A smaller number reported that they had committed to the charter by completing the on-line quiz. This may reflect the fact that this was only made available within the last two months of the implementation process or that there was insufficient opportunity or motivation to do so.

With only a small number of responses from residents, it is difficult to draw any conclusions from their responses. They seemed less confident in the ability of the charter to improve the quality of their care. However, this may reflect the fact that they are already satisfied with the care they receive. A clear limitation of this study is that many of the questions used within this survey are predicated on the basis that the current practice could be improved. If this is not perceived to be the case by respondents, then they are unlikely to expect further improvement in practice and resident care to be realized. One respondent reported that, although she had not seen the charter, she was confident in most of the areas questioned that supports this assertion.

However, although homes were approached by senior and respected individuals within each area, only slightly more than half who agreed attended the launch event and these were more senior staff (junior nursing staff did not attend) and less than 50% returned completed questionnaires at the end of the pilot despite the best efforts of the research team. With such a low response rate, the representativeness of the responses within the returned questionnaires is unknown and it may be that non-responders would respond differently. As a pilot, we were, however, more interested in what could be learned from the process and believed that we received sufficient responses to enable us to plan for national roll-out.

The charter website was designed to be educational (and included case studies) and training was provided at the launch events with a certificate provided in recognition of successfully completing the on-line quiz/assessment. No other incentives were provided to encourage the ongoing use of the charter. Staff will only use the charter standards if they are required/encouraged to by the care home. Care homes themselves will need encouragement and this typically comes via the regulatory framework. Consequently, involvement of quality assurance bodies such as the Care Quality Commission in England [37] may be necessary to coerce changes in practice.

Care homes cannot act alone and will need support from other health professionals. General practitioners, speech and language therapists, and pharmacists who have their own professional and employment priorities may require incentives through commissioning to ensure that access to their expertise and support is available from the charter launch through the implementation and evaluation.

## 5. Conclusions

An evidence-based and expert-informed best practices charter for residents and caregivers in care homes to improve medication practices especially in those with dysphagia was designed and successfully tested across the UK. Participants and respondents were overwhelmingly positive regarding the structure of the launch events, the usefulness of the content within the supporting website, and the potential effect of the charter on resident care.

Attendance at launch events and the questionnaire response could have been significantly improved, as could the perceived overall effectiveness of the charter. Areas of implementation, which were not addressed within this process and may increase its eventual effectiveness, related to the coercion of homes to improve the quality of care via quality assurance agencies and environmental restructuring and local coercion by care home managers and owners to encourage care home staff engagement and implementation. Access to other healthcare professionals external to the home is required to enable effective charter implementation and this may be achieved through external commissioning of their services.

Further evaluation of the clinical outcomes of charter implementation in a larger sample of long-term care facilities is warranted.

## Figures and Tables

**Table 1:** Care Home Charter (www.carehomecharter.org).

Resident Charter	Care Home Worker Charter
When I am staying in a care home, I expect people responsible for my care to:	As a professional working in a care home I must have the requisite knowledge and skills to:
1. Actively involve me in decisions about my medicine	1. Identify and respect the resident’s wishes and beliefs about medicine
2. Help me make shared decisions about my medicine	2. Involve the resident and/or those important to them to make shared decisions about medication
3. Regularly monitor and review my medicine	3. Ensure the resident I care for will have a regular medication optimization review
4. Ensure that medicines are administered in a form/route appropriate to my needs/abilities	4. Assess, monitor, administer, and review medication to ensure that the resident receives medication in an appropriate form and route
5. Ensure that I am not given medicine against my wishes	5. Only administer medicine in line with national and covert medication administration policy and the guidance of the court of protection
6. Respect the advance decisions or directives I make regarding refusing my medicine	6. Ensure an advance care plan, which includes medication, is in place for the resident with a regular review when their condition changes
7. Ensure that medicine is not given to me hidden in my food unless it is in my best interests and all legal requirements have been met	7. Work with other members of the multi-disciplinary team to ensure that the resident’s medication needs are met
8. Examine my mouth to ensure that my oral health needs are being met	8. Ensure that oral and dental care is provided for residents
9. Recognize when I am unable to swallow safely	9. Recognize and manage swallowing problems to ensure that appropriate referrals are made

**Table 2:** Respondent summary of how the charter and website has been implemented and the perceived effect on staff and relatives/residents.

Question	Response		
	Junior Staff (%)	Senior Staff (%)	Resident/Relative (%)
Copy of charter available to you (Senior n = 18. Junior n = 18)	17 (94.4)	16 (89.9)	
Did you access the website (Senior n = 17, Junior n = 17)	16 (94.1)	8 (47.1)	
Did you commit to the charter using the on-line quiz (Senior n = 15, Junior n = 15)	7 (46.7)	3 (20)	
**Which of the following tools from the website have you used?**	**N = 16**	**N = 8**	
Tips for embedding the charter in your home	11 (68.8)	5 (62.50	
On-line course on Dysphagia: Swallowing difficulties and medicines	7 (43.8)	2 (25)	
Guidelines in practice	11 (68.8)	4 (50)	
Dysphagia identification checklist	10 (62.5)	5 (62.5)	
Care plan reminder template	10 (62.5)	3 (37.5)	
Audit checklist	9 (56.3)	5 (62.5)	
**Which of the Resources from the website have you used?**	**N = 16**	**N = 16**	
NHS advice on swallowing difficulties	10 (62.5)	6 (75.0)	
Learning advice on disabilities for people with swallowing difficulties	7 (43.8)	5 (62.5)	
NEWT guidelines for the administration of medication to patients with enteral feeding tubes or swallowing difficulties	4 (25.0)	1 (12.5)	
Swallowing difficulties website	11 (68.8)	6 (75.0)	
Prescribing decision support website	6 (37.5)	3 (37.5)	
Advanced decisions and general information	7 (43.8)	2 (25.0)	
General medicine-related support and advice	8 (50.0)	4 (50.0)	
**Overall usefulness of the charter within the role**	**N = 17**	**N = 15**	**N = 7**
Very negative effect	0	0	
Negative effect	0	0	
No effect	5.9	0	
Useful	58.8	66.7	
Very useful	35.3	33.3	
**Overall usefulness of the charter in improving the quality of care for residents (%):**	**N = 17**	**N = 15**	**N = 7**
Very negative effect	0	0	0
Negative effect	0	0	0
No effect	0	50.0	50.0
Useful	80.0	33.3	33.3
Very useful	20.0	16.7	16.7
